# Hemichordates’ diffuse “skin brain” shows unexpected complexity

**DOI:** 10.1371/journal.pbio.3002312

**Published:** 2023-09-20

**Authors:** Alexandra Kerbl, Gáspár Jékely

**Affiliations:** Centre for Organismal Studies (COS), University of Heidelberg, Heidelberg, Germany

## Abstract

Hemichordates, close relatives of chordates, have a nervous system that is chordate-like in its patterning but their neural architecture remains unexplored. This Primer explores a new study in PLOS Biology that reveals an unexpected neuroanatomical complexity in these animals.

## Teaser

Hemichordates are close relatives of chordates. Their nervous system patterning is chordate-like, but their neural architecture remains unexplored. A new study in *PLOS Biology* reveals an unexpected neuroanatomical complexity in these animals, also informing chordate origins.

**Fig 1:**
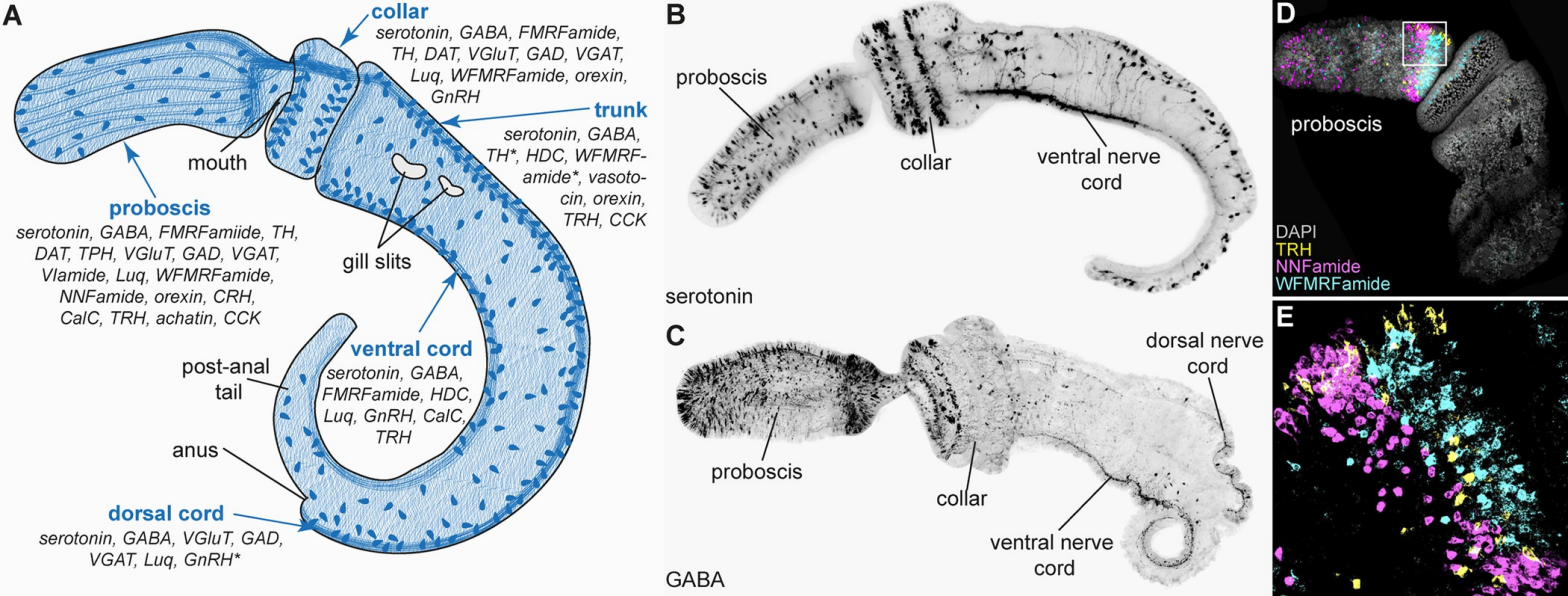
Molecular atlas of *Saccoglossis kowalevskii* (Hemichordata). (**A**) Schematic drawing of a *S*. *kowalevksii* late juvenile and its nervous system. Although the nerve plexus looks diffuse, Andrade Lopéz and colleagues identified several distinct neuron classes with regionalised distribution. (**B**) Serotonin- and (**C**) GABA-like immunoreactivity mark neurons with representative projection patterns. Cell bodies scattered throughout the proboscis and project posteriorly and posterior neurons project anterior. (**D**, **E**) The base of the proboscis harbours almost all identified neuron types, often enriched dorsally and in close proximity to another, as shown by the colocalization of 3 neuropeptides (with DAPI staining as nuclear reference) (from Andrade Lopéz and colleagues). Abbreviations: CalC, calcitonin; CCK, cholecystokinin; CRH, corticotropin-releasing hormone; DAT, dopamine transporter; GAD, glutamate decarboxylase; GnRH, gonadotropin-releasing hormone; HDC, histidine decarboxylase; Luq, luqin (RNamide); TH, tyrosine hydroxylase; TPH, tryptophan hydroxylase; TRH, thyrotropin-releasing hormone; VGAT, GABA transporter; VGluT, glutamate transporter. *Weak immunoreactivity mainly in the anterior part of the trunk or around the gill slits.

Acorn worms or enteropneusts (belonging to the phylum Hemichordata) are rather inconspicuous worms living as adults in muddy or sandy sediments in the oceans. Their body is fully ciliated and consists of an elongated, muscular proboscis followed by a narrow, constricted neck region and a rather short, fleshy collar, which continues into a worm-like trunk (Fig 1). Due to their phylogenetic position as members of the sister group to the chordates, hemichordates were often studied as an outgroup to understand chordate origins. They also have received special attention because of their bilateral symmetry, which makes comparisons to chordates more straightforward, as opposed to the colonial pterobranchs (also hemichordates) or pentaradial echinoderms.

Molecular similarities in the expression of developmental genes have revealed striking similarities in nervous system regionalisation between hemichordates and chordates [1,2]. Despite this, the hemichordate nervous system does not show the concentration, regionalization, and specification observed in chordates and is often referred to as a “nerve net.” A new study by Andrade Lopéz and colleagues presents new high-resolution neuroanatomical data on the hemichordate acorn worm *Saccoglossus kowalewskii* and reveals the regionalised distribution and morphology of several neuron classes including intricate long-range projections [3]. The paper demonstrates that the acorn worm nervous system is anything but an “unstructured nerve plexus” and that there is a hidden neuroanatomical complexity still to be uncovered in these understudied animals.

The diffuse organisation of the nervous system of acorn worms (Fig 1) stands in contrast to the high level of concentration seen in chordates, fuelling a debate about the evolutionary origins of centralised nervous systems [4–6]. One opinion favoured the independent origin of centralisation in chordates and protostomes (including insects and annelids), with hemichordates retaining an ancestral net-like organisation also seen in cnidarians [5]. Others argued for a centralised nervous system in the last bilaterian common ancestor that later became partially diffuse in hemichordates [6]. More detailed studies on adult acorn worms have taken the edge off this debate and revealed substantial centralisation in the dorsal and ventral cords, with the dorsal cord even forming an internalised chordate-like neural tube in the collar region [7]. However, the nature of the neural net and whether it can be homologised to the nerve net in cnidarians or is more akin to a peripheral nervous system has remained unclear.

Another puzzle that emerged from molecular studies of nervous system patterning in hemichordates related to the disparity between molecular coordinates and the underlying anatomy. While the expression of anterior–posterior patterning genes and the position and molecular makeup of brain signalling centres—such as the zona limitans intrathalamica and the isthmic organiser—is strikingly similar between hemichordates and chordates, there is no apparent similarity in the neuroanatomy that develops [5,8–10].

What has been lacking to resolve these long-standing questions is more details of the anatomy of the hemichordate nervous system. Our knowledge of hemichordate neuroanatomy has still largely been based on classic investigations with a resolution that was not sufficient to reveal many individual neurons, neurite paths, or synapses [11]. Andrade Lopéz and colleagues [3] have now utilised a comprehensive set of tools to provide the most complete description of neuroanatomy in the acorn worm *Saccoglossus kowalewskii* to date. Their work combines conventional histological staining, labelling for neurotransmitter markers, dye filling, and the use of transgenic reporters. These approaches allowed them to characterise the detailed anatomy of several neuron classes, including the location of their soma, the morphology of dendrites and axons, and the distribution of putative synapses. One key finding was that several neuron types exhibit strong anteroposterior and dorsoventral concentrations. For example, several conserved neuropeptides show strong expression at the dorsal proboscis base, suggesting the presence of a neuroendocrine centre possibly releasing neuropeptides into the underlying coelomic fluid or circulatory system. Andrade Lopéz and colleagues also identified neuron types that distinguish the 2 nerve cords in the trunk. Glutamatergic neurons flank the dorsal cord, whereas histaminergic neurons mark the ventral cord, supporting early observations that the dorsal and ventral cords have different functions [11].

Neuronal labelling also revealed the detailed cell morphology of several neuron classes [3]. The authors found that the majority of the neurons they labelled were sensory neurons. These have a flask shape, with an apically projecting dendrite, often continuing in a sensory cilium. Some of these, like serotonergic sensory neurons, tiled the entire proboscis and projected posteriorly. Individually labelled neurons showed several varicosities (indicative of synapses) along their axons. Some neurons had very long projections spanning almost the entire body.

Overall, the data suggest a hitherto unseen regionalization and complexity of the hemichordate nervous system. It is likely that the complex molecular regionalisation observed by gene expression analyses [5,8] patterns an underlying distribution of specific neuronal cell types, even if this does not manifest in a macroscopic morphological pattern. Regarding the nerve net, the authors found no evidence that the acorn worm nerve plexus has an organisation like that of the simple nets of cnidarians, with neurons directly synapsing on their immediate neighbours [3]. This cautions against homologising the cnidarian, hemichordate, or other nerve plexuses as “nerve nets.”

The rich anatomical resource also helps to reinterpret early observations of acorn worm behaviour [11]. The worms move by the combined action of cilia and muscles. They can burrow in the sediment through the peristaltic action of the proboscis. They also react to light, mechanical, or chemical stimuli. Mechanical stimulation of the trunk can lead to ciliary arrest and reversal. The entire body appears photosensitive, as dissected pieces can contract on illumination [11]. The tiling of the body by various sensory neurons with long-range projections suggests a diffuse sensory-motor organisation, controlling muscles and ciliated cells, enabling the pieces to behave as the whole. Autonomous nervous activity seems to be centred in the proboscis, which is also the most active region and affects the movement of the rest of the body [11]. This agrees with the new anatomical data, which identify the base of the proboscis as a likely integrative centre [3].

The study by Andrade Lopéz and colleagues revealed a hidden complexity of nervous system organisation in hemichordates hinting at a complex bilaterian brain under their skin with specific connections formed by a large number of neuron types with specific functions. This should attract new attention to these enigmatic animals and stimulate work on their behaviour, neuronal function, and wiring (connectome), also helping to reconstruct chordate origins.
